# Association of baseline D-dimer with adverse outcomes after percutaneous coronary intervention in patients with coronary heart disease: A meta-analysis

**DOI:** 10.1097/MD.0000000000046910

**Published:** 2026-01-23

**Authors:** Huanlun Li, Yun Liang, Zhichao Yuan, Lihua Lu, Tong Liao

**Affiliations:** a Department of Cardiovascular Medicine, Dongguan Dalong Hospital, Dongguan, Guangdong Province, China.

**Keywords:** D-dimers, meta-analysis, myocardial infarction, percutaneous coronary intervention

## Abstract

**Background:**

To investigate the association between baseline D-dimers (DD) and adverse outcomes after percutaneous coronary intervention (PCI) in patients with coronary heart disease (CHD) by meta-analysis.

**Methods:**

Relevant literature was obtained by searching PubMed, Web of Science, Cochrance Library, and Embase until November 2024. The hazard ratio (HR) and 95% confidence interval (CI) were pooled for each study using either a fixed or random-effects model. The clinical outcomes analyzed were all-cause mortality, cardiovascular mortality, major adverse cardiovascular events (MACE), and revascularization.

**Results:**

A total of 10 articles were included in this meta-analysis. The results of meta-analysis showed that high baseline DD levels were associated with an increased risk of all-cause mortality (HR = 2.35, 95% CI: 1.78–3.10, *P* < .001), cardiovascular mortality (HR = 2.94, 95% CI: 1.99–4.33, *P* < .001), and MACE (HR = 1.74, 95% CI: 1.25–2.42, *P* = .001) after PCI in patients with CHD. However, no association was found between baseline DD level and revascularization risk (HR = 1.02, 95% CI: 0.76–1.37, *P* = .893).

**Conclusion:**

Baseline DD level can predict adverse clinical outcomes after PCI in patients with CHD. High baseline DD levels were significantly associated with an increased risk of all-cause mortality, cardiovascular mortality, and MACE.

## 1. Introduction

Coronary atherosclerotic heart disease refers to heart disease caused by coronary artery stenosis or blockage of blood vessels, or myocardial ischemia, hypoxia or necrosis due to functional changes of coronary arteries, collectively referred to as coronary heart disease (CHD), also known as ischemic heart disease.^[1,2]^ Revascularization is a very effective measure for the treatment of CHD. Although it cannot completely cure CHD, it can improve clinical symptoms, the quality of life and survival rate of patients.^[3]^ Percutaneous coronary intervention (PCI) is the main means of treatment for CHD patients. Previous studies have revealed that GRACE, PURSUIT, and TIMI risk scores can predict the long-term prognosis of post-PCI in CHD patients.^[4]^ Intravascular ultrasound, optical coherence tomography, and fractional flow reserve, etc, can also predict its long-term forecast.^[5–7]^ However, these methods have limitations, such as invasive, high cost, complex operation and low accuracy. Therefore, it is particularly essential to identify a simple process to predict the long-term prognosis of post-PCI accurately.

In recent years, the role of activation of coagulation and fibrinolytic systems in the pathogenesis and prognosis of CHD has received extensive attention.^[8,9]^ D-dimer (DD) is a specific degradation product produced when fibrinolytic protease acts on fibrin. Nonetheless, when pathological changes occur, that is, when coagulation occurs in the body, fibrin will cross-link and trans synthesize fibrin under the action of thrombin. Moreover, the fibrinolytic system will be activated and fibrin will then be cleaved to form various types and large amounts of fragments.^[10]^ The broken fragments containing D fragments are connected by the r chain to form DD. As the level of DD increases, it marks the formation of blood clots in the vascular circulatory system. It can be seen that the level of DD can be used as one of the sensitive markers to judge whether there is thrombosis in the blood vessels.^[11]^ Studies on patients with different diseases have found that the increase in DD level is associated with a higher risk of all-cause mortality.^[12,13]^ High DD level is an independent risk factor for patients with stable CHD and can predict long-term mortality, cardiovascular events and the occurrence of cancer.^[14]^

Although many studies have investigated the association between baseline DD levels and adverse outcomes after PCI in patients with CHD, the results remain controversial. Therefore, in this study, meta-analysis was used to explore the correlation between baseline DD levels and adverse outcomes after PCI in patients with CHD, in order to provide evidence-based medical evidence for clinical prediction of prognosis after PCI in patients with CHD.

## 2. Methods

### 2.1. Literature screening

This study was approved by the Ethics Committee of Dongguan Dalong Hospital. The relevant literature was obtained by searching PubMed, Web of Science, Cochrance Library, and Embase until November 2024. The keywords were DD, CHD, myocardial infarction, PCI, etc. The search language was limited to English. Besides, the references of relevant reviews for literature that might meet the requirements were checked. During this process, if any differences emerged, they would be resolved through discussion.

### 2.2. Inclusion and exclusion criteria

Inclusion criteria: prospective or retrospective cohort study; the subjects were patients with CHD undergoing PCI; the study evaluated the association between baseline DD levels and outcomes of PCI in patients with CHD, providing direct reports in the study or sufficient information to calculate hazard ratio (HR) or relative risk with 95% confidence interval (CI).

Outcome indicators: all-cause mortality, cardiovascular mortality, major adverse cardiovascular events (MACE), and revascularization after PCI in patients with CHD.

Exclusion criteria: reviews, reviews, conference papers, animal, and cell studies; duplicate studies or data; incomplete literature and lack of sufficient research data; no clinical outcome reported.

### 2.3. Literature quality evaluation

The included cohort studies were evaluated according to the Newcastle–Ottawa Scale (NOS).^[14,15]^ In order to determine literature quality, 2 independent researchers extracted relevant information and developed grading criteria for literature quality. Differences between 2 researchers were resolved through discussion or by a third author. The total NOS score was 9 points. Studies with NOS scores of at least 6 were considered to be of high quality.

### 2.4. Data extraction

The 2 researchers extracted the data independently and cross-checked them to ensure the reliability of the information. The following data were extracted: 1st author, years of publication, countries of study subjects, sample size, age, cutoff value, and outcome measures.

### 2.5. Statistical analysis

Stata 15.0 software was used for meta-analysis and quantitative synthesis of data. The Chi-square test and *I*^2^ statistics were applied to analyze the statistical heterogeneity of the included literatures. If *P* ≥ .1 and *I*^2^ < 50%, no statistical heterogeneity existed, and then the fixed-effect model was used for analysis. If statistical heterogeneity was found (*P* < .1, or *I*^2^ ≥ 50%), a random-effects model was used. HR and 95% CI were used as effect indicators. If the number of included references was >5, Egger test was selected to detect publication bias. Sensitivity analysis was conducted to verify the robustness of the results. *P* < .05 was considered statistically significant.

## 3. Results

### 3.1. Literature search results

**Figure 1.**
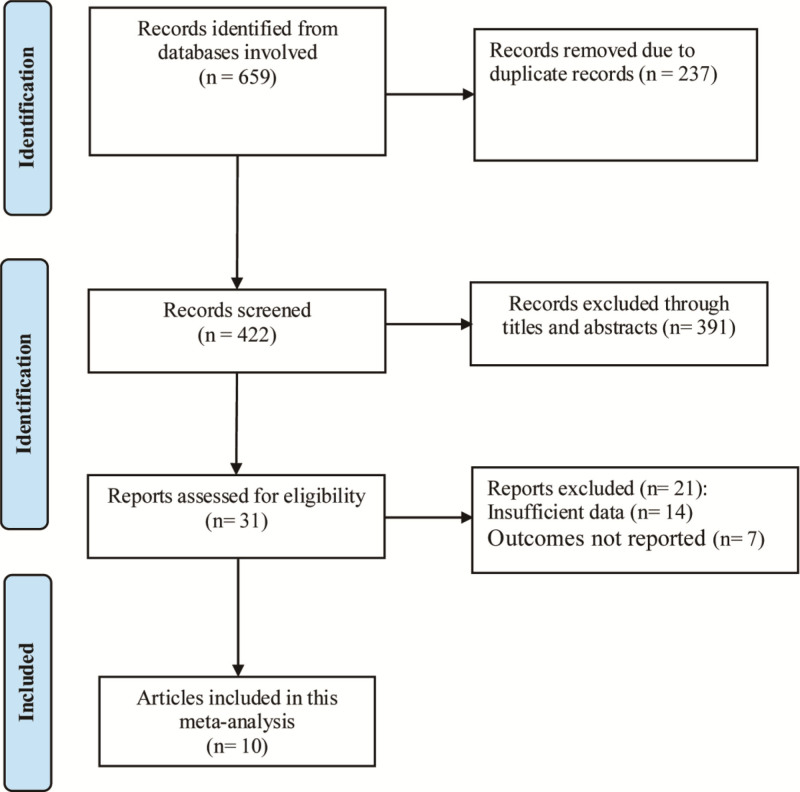
Detailed flowchart of literature screening.

After the initial search, a total of 993 relevant entries were obtained. After preliminary review of the titles and abstracts, 26 records remained. After downloading the full text of these records for further screening, a total of 10 trials^[16–25]^ were finally included in this meta-analysis (Fig. 1), involving 28,537 patients. The basic characteristics of the included literatures are shown in Table 1. The NOS scores of the included records were all >5, indicating that the studies included were all high quality.

**Table 1:** Basic characteristics of included studies.

Study	Year	Country	Patients	Ages	Cutoff value	NOS	Outcomes
Akgul	2013	Turkey	453	55.6 ± 12.4	0.72	7	①②③④
Erkol	2014	Turkey	569	56 ± 12	0.41	8	①②③④
Sarli	2014	Turkey	266	64 ± 10	544 μg/mL	8	③
Huang D	2020	China	1165	62.5 ± 11.6, 67.9 ± 12.1	0.8 mg/L	8	③
Zhao XY	2020	China	8565	55.5 ± 9.3, 61.1 ± 10.4	0.28 μg/mL	7	①②
Zhou Q	2020	China	872	63.7 ± 12.7	Median	8	①④
Chen RZ	2022	China	3972	59 ± 11.9	NR	8	①②③
Kurosawa	2022	Japan	1440	64.6 ± 11.3, 69 ± 10.8, 72.4 ± 10.8	1.2	8	①②
Li JW	2023	China	10,724	58.4 ± 10.3	Median	8	①②
Zhang KP	2024	China	511	61.7 ± 13.9, 60.3 ± 15	NR	8	③

①: all-cause mortality; ②: cardiovascular mortality; ③: major adverse cardiovascular events; ④: revascularization; NR = not reported.

### 3.2. Meta-analysis results

#### 3.2.1. Heterogeneity analysis

**Figure 2.**
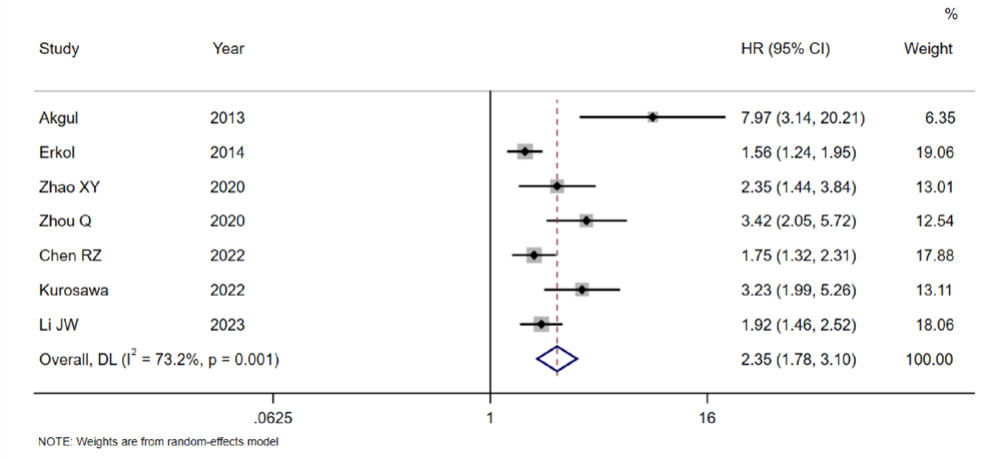
Forest plot of D-dimer on all-cause mortality after PCI in coronary heart disease. PCI = percutaneous coronary intervention.

**Figure 3.**
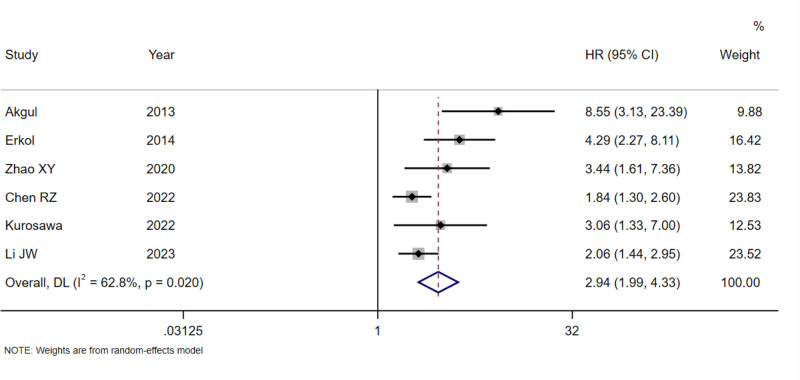
Forest plot of D-dimer on cardiovascular mortality after PCI in coronary heart disease. PCI = percutaneous coronary intervention.

**Figure 4.**
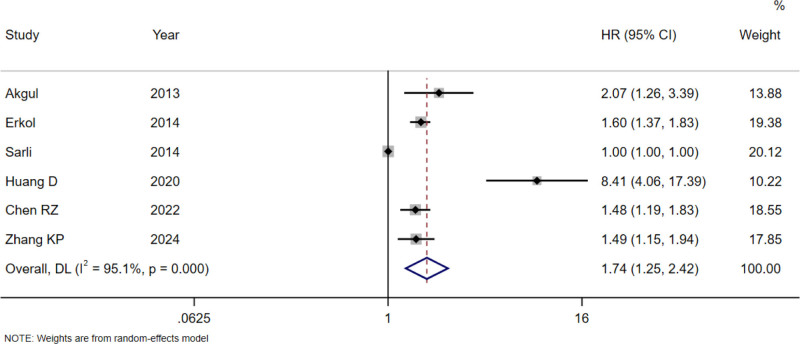
Forest plot of D-dimer on MACE after PCI in coronary heart disease. MACE = major adverse cardiovascular events, PCI = percutaneous coronary intervention.

**Figure 5.**
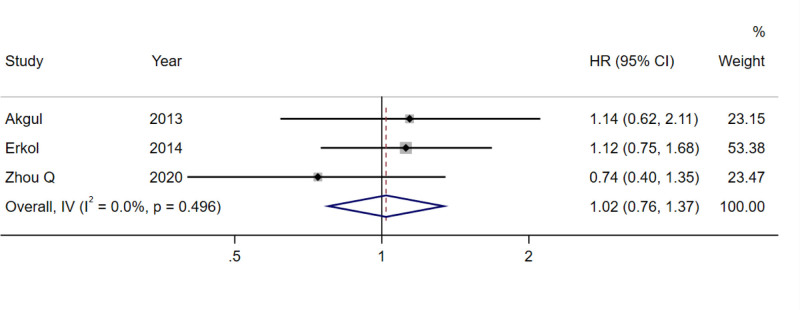
Forest plot of D-dimer on revascularization after PCI in coronary heart disease. PCI = percutaneous coronary intervention.

Significant heterogeneity was found in the pooled analyses for all-cause mortality (*I*^2^ = 73.2%, *P* = .001; Fig. 2), cardiovascular mortality (*I*^2^ = 62.8%, *P* = .020; Fig. 3), and MACE (*I*^2^ = 95.1%, *P* < .001; Fig. 4), so the random-effects model was used. In the analysis of revascularization (Fig. 5), no heterogeneity was seen (*I*^2^ = 0.0%, *P* = .496), so the fixed-effects model was applied for data consolidation.

#### 3.2.2. Analysis of adverse outcomes

Seven, 6, 6, and 3 records were included in the combined analysis of all-cause mortality (Fig. 2), cardiovascular mortality (Fig. 3), MACE (Fig. 4), and revascularization (Fig. 5), respectively. The results of meta-analysis exhibited that high baseline DD levels were significantly associated with an increased risk of all-cause mortality (HR = 2.35, 95% CI: 1.78–3.10, *P* < .001), cardiovascular mortality (HR = 2.94, 95% CI: 1.99–4.33, *P* < .001), and MACE (HR = 1.74, 95% CI: 1.25–2.42, *P* = .001) in patients with CHD after PCI. The differences were statistically significant. However, no association was found between baseline DD level and revascularization risk (HR = 1.02, 95% CI: 0.76–1.37, *P* = .893), with no statistically significant differences.

### 3.3. Publication bias detection

Regarding all-cause mortality, cardiovascular mortality, and MACE, the *P*-values of Egger test were < .001, .012, and .004, respectively, suggesting publication bias in this review.

### 3.4. Sensitivity analysis

**Figure 6.**
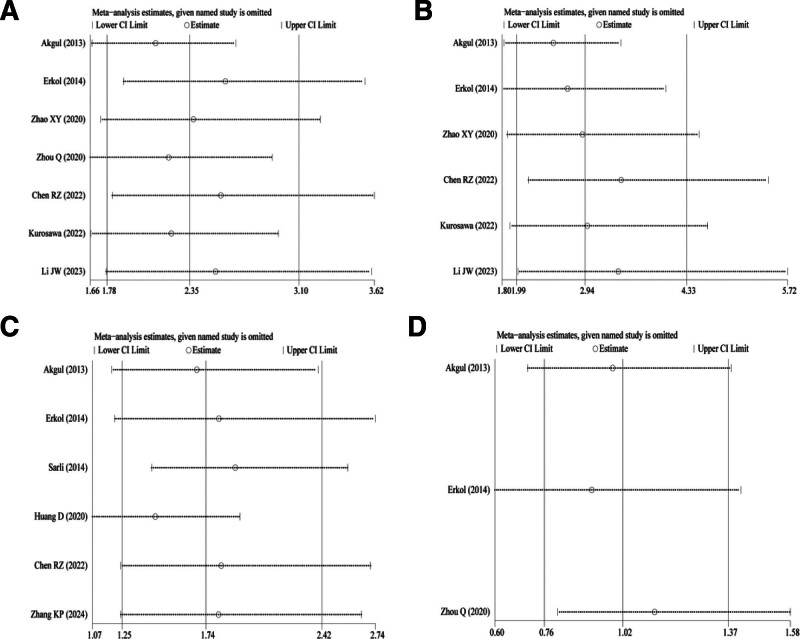
Sensitivity analysis results of (A) all-cause mortality, (B) cardiovascular mortality, (C) MACE, and (D) revascularization. MACE = major adverse cardiovascular events, PCI = percutaneous coronary intervention.

In the sensitivity analysis of all-cause mortality, cardiovascular mortality, MACE, and revascularization (Fig. 6A–D), no significant change was found in results after pooled analysis when excluding each record, indicating that the conclusions of this meta-analysis are robust.

## 4. Discussion

In patients with CHD, cardiovascular disease is damaged for a long time, which will reduce the diastolic function of the heart, cause the abnormal structure of the coronary artery lumen, reduce the contractility of the myocardium, and damage myocardial cells. Coronary heart disease is prone to complications of various serious cardiovascular diseases (heart failure, ventricular aneurysm, etc), and thus has a high fatality rate.^[26]^ Currently, many biomarkers are used to assess the prognosis of patients with CHD.^[27,28]^ However, some are relatively expensive or difficult to apply in clinical practice. This study used meta-analysis to explore the correlation between baseline DD and adverse outcomes in CHD patients after PCI, finding that DD could be used to predict adverse clinical outcomes in CHD patients after PCI, such as all-cause mortality, psychogenic mortality, and MACE. The inclusion of DD in clinical prediction models may significantly improve the ability to predict death in patients with CHD after PCI, which can help identify patients at higher risk of death.

DD is a specific degradation product of cross-linked fibrin and is mainly produced when the body is in a hypercoagulable or hyperfibrinolytic state. Elevated DD levels indicate enhanced coagulation activation and secondary fibrinolysis, and numerous studies have shown that higher DD levels are associated with an increased risk of cardiovascular adverse events.^[29,30]^ When myocardial ischemia occurs, endothelial injury triggers activation of the coagulation cascade and thrombin generation, leading to fibrin formation and blood hypercoagulability. At the same time, compensatory activation of the fibrinolytic system results in degradation of fibrin into DD fragments.^[31,32]^ In addition to reflecting thrombosis, recent evidence suggests that DD also links inflammation to coagulation–fibrinolysis imbalance. Proinflammatory cytokines such as interleukin-6 and tumor necrosis factor-α upregulate tissue factor expression on endothelial cells and monocytes, thereby activating the extrinsic coagulation pathway. Meanwhile, inflammation suppresses fibrinolysis by increasing plasminogen activator inhibitor-1 and reducing tissue plasminogen activator activity. This combined effect promotes microthrombosis, endothelial dysfunction, and plaque instability, resulting in a sustained elevation of DD levels and adverse cardiovascular outcomes. Following PCI, vascular injury and stent implantation further enhance local inflammation, platelet activation, and thrombin generation, which may contribute to no-reflow, in-stent restenosis, microvascular obstruction, and recurrent ischemic events. Previous studies have reported that elevated DD levels independently predict no-reflow within 2 to 7 days after PCI in ST-elevation myocardial infarction patients.^[33]^ Similarly, in the long-term prognosis of patients with acute coronary syndrome, higher baseline DD levels were associated with an increased risk of all-cause mortality.^[34]^ Therefore, DD not only serves as a marker of coagulation activation but may also reflect systemic inflammatory burden and thrombotic activity, making it a valuable indicator for risk stratification in patients with CHD undergoing PCI.

In recent years, more and more clinical trials have demonstrated that DD has key value in the pathogenesis of CHD and the prediction of adverse events. One study reported that the higher the level of DD in patients, the greater the risk of blood clots.^[35]^ This also explains the correlation between DD and CHD. There is evidence that a positive correlation exists between DD levels and atherosclerotic plaque vulnerability.^[36]^ Besides, plasma DD levels were associated with local unstable plaque formation in coronary arteries. The level of DD not only increased in patients with acute coronary syndrome, but also significantly increased in patients with recurrent myocardial infarction.^[37]^

After a comprehensive search and strict inclusion criteria, 10 articles were included in this meta-analysis. The pooled analysis presented that an increase in baseline DD was significantly associated with an increased risk of all-cause mortality, heart rate mortality, and MACE in patients with CHD after PCI. However, no significant association was found between increased DD and the risk of revascularization. In order to verify the robustness of the findings of this study, we excluded each article one by one, conducting a combined analysis again. The results showed that there was no significant change between the obtained results and the initial results after excluding any reference. Therefore, the findings obtained in this meta-analysis had good robustness. Notably, in terms of all-cause mortality, cardiovascular mortality, and MACE, the *P*-values of Egger test were all <0.05, indicating publication bias in this meta-analysis.

Inevitably, this meta-analysis had several limitations. First, a possibility of publication bias might exist in this study. Publication bias was evaluated using Egger test, which is considered more sensitive than Begg test when more than 5 studies are included. Although Begg test was not performed separately, sensitivity analyses demonstrated that the exclusion of any single study, including those with smaller sample sizes, did not materially change the pooled estimates, suggesting the robustness of our findings. Second, fewer records were included and the sample size was relatively small, which might influence the robustness of the conclusions. Third, only studies published in English were eligible for this study, which might introduce language-related publication bias. Fourth, significant heterogeneity was observed in the pooled analyses of all-cause mortality, cardiovascular mortality, and MACE. To minimize the impact of heterogeneity, a random-effects model was applied for these outcomes, whereas a fixed-effects model was used for revascularization due to the absence of heterogeneity. Although subgroup analyses (such as ACS vs stable CHD, DD cutoff values, follow-up duration, and regional differences) and meta-regression could have been helpful to further explore the sources of heterogeneity, these methods were not feasible because of the limited number of available studies and insufficiently reported study-level data. Fifth, all the included studies were observational cohort studies rather than randomized controlled trials; therefore, residual confounding factors, such as treatment strategy and baseline clinical characteristics, may still affect the results despite multivariable adjustments in the original studies.

In conclusion, baseline DD levels can predict adverse clinical outcomes after PCI in patients with CHD. High baseline DD levels were significantly associated with an increased risk of all-cause mortality, cardiovascular mortality, and MACE. However, given the limitations of this meta-analysis, more high-quality trials are warranted to verify the findings of this study in the future.

## Author contributions

**Conceptualization:** Huanlun Li, Yun Liang, Zhichao Yuan, Lihua Lu, Tong Liao.

**Data curation:** Huanlun Li, Yun Liang, Zhichao Yuan, Lihua Lu, Tong Liao.

**Formal analysis:** Huanlun Li, Yun Liang, Zhichao Yuan, Lihua Lu, Tong Liao.

**Funding acquisition:** Tong Liao.

**Investigation:** Tong Liao.

**Writing – original draft:** Huanlun Li, Yun Liang, Zhichao Yuan, Lihua Lu, Tong Liao.

**Writing – review & editing:** Huanlun Li, Yun Liang, Zhichao Yuan, Lihua Lu, Tong Liao.
